# Supplementary material for: Associations between Animal Welfare Indicators and Animal-Related Factors of Slaughter Cattle in Austria
Source: Animals (Basel). 2022 Mar 5;12(5):659. doi: 10.3390/ani12050659 (PMC8909719; doi:10.3390/ani12050659)
Supplement: Supplementary file 1 [file animals-12-00659-s001.zip › animals-1605345-supplementary.pdf]

animals

MDPI

Supplementary Material

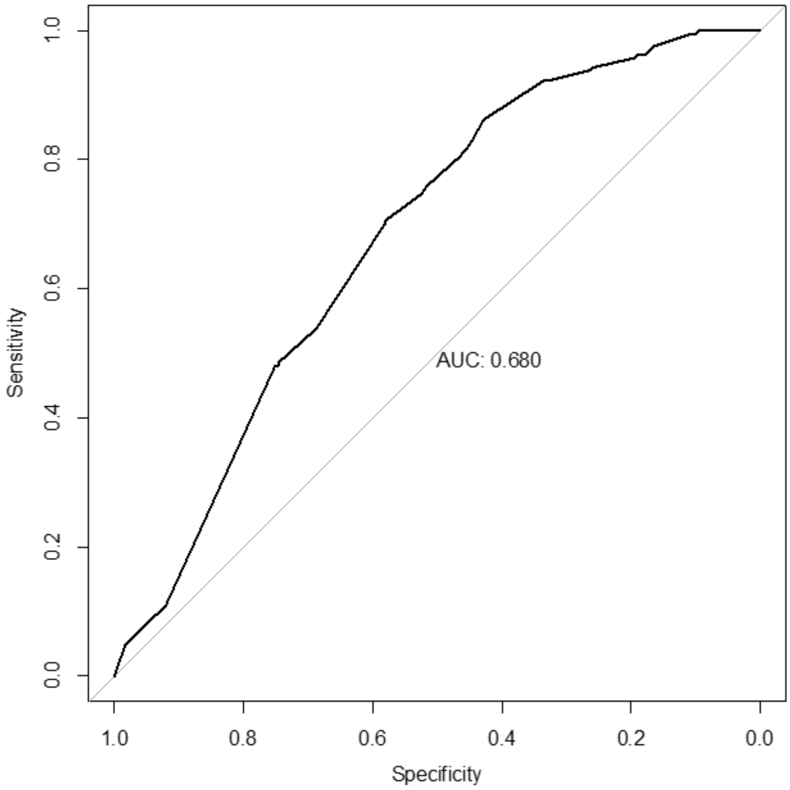

Figure S1. Receiver Operating Characteristics (ROC) graph of the final mixed binomial logistic model.

	Cross Validation	Confusion Matrix
**Accuracy**	**64.4%**	**68.0%**
**Sensitivity**	**51.9%**	**56.0%**
**Specificity**	**75.7%**	**79.5%**
**Positive prediction**	**65.8%**	**71.1%**
**Negative prediciton**	**63.6%**	**66.8%**

AUC= Area under the curve.

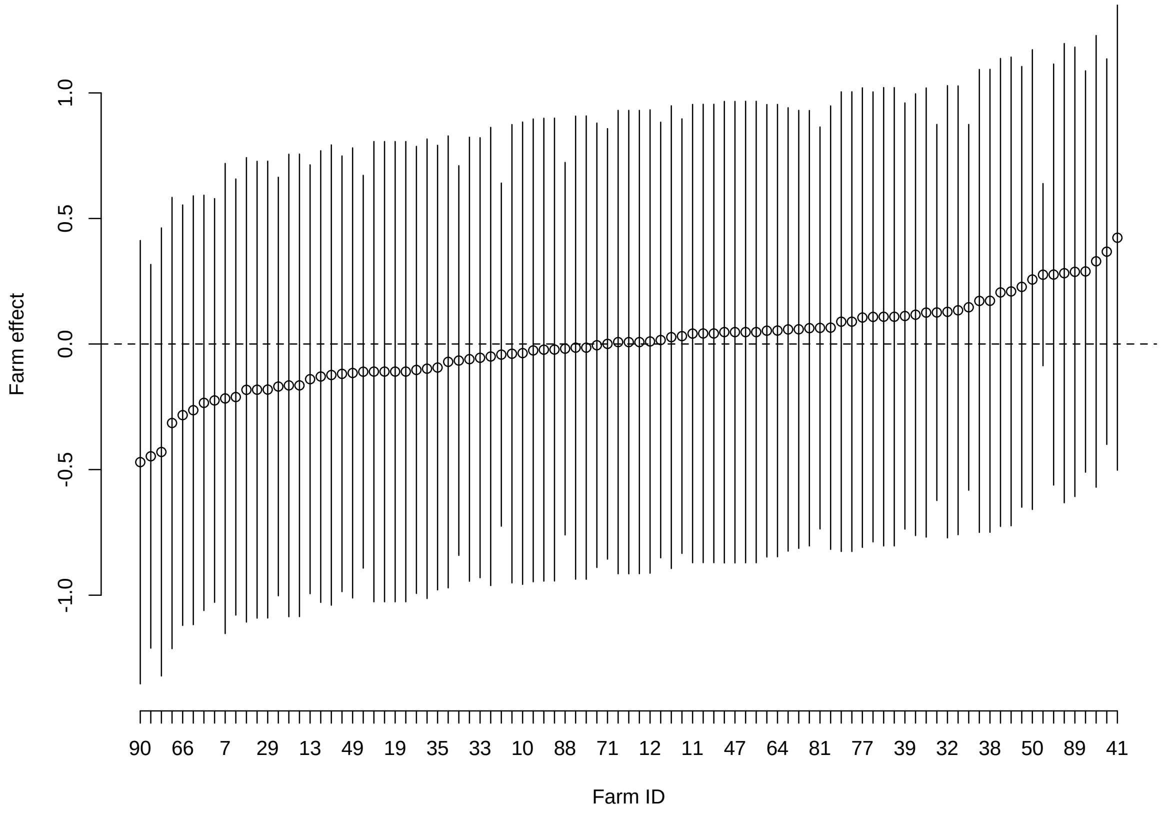

Figure S2. Estimated random intercepts and 95% prediction intervals for random effect farm based on the cumulative link mixed model.

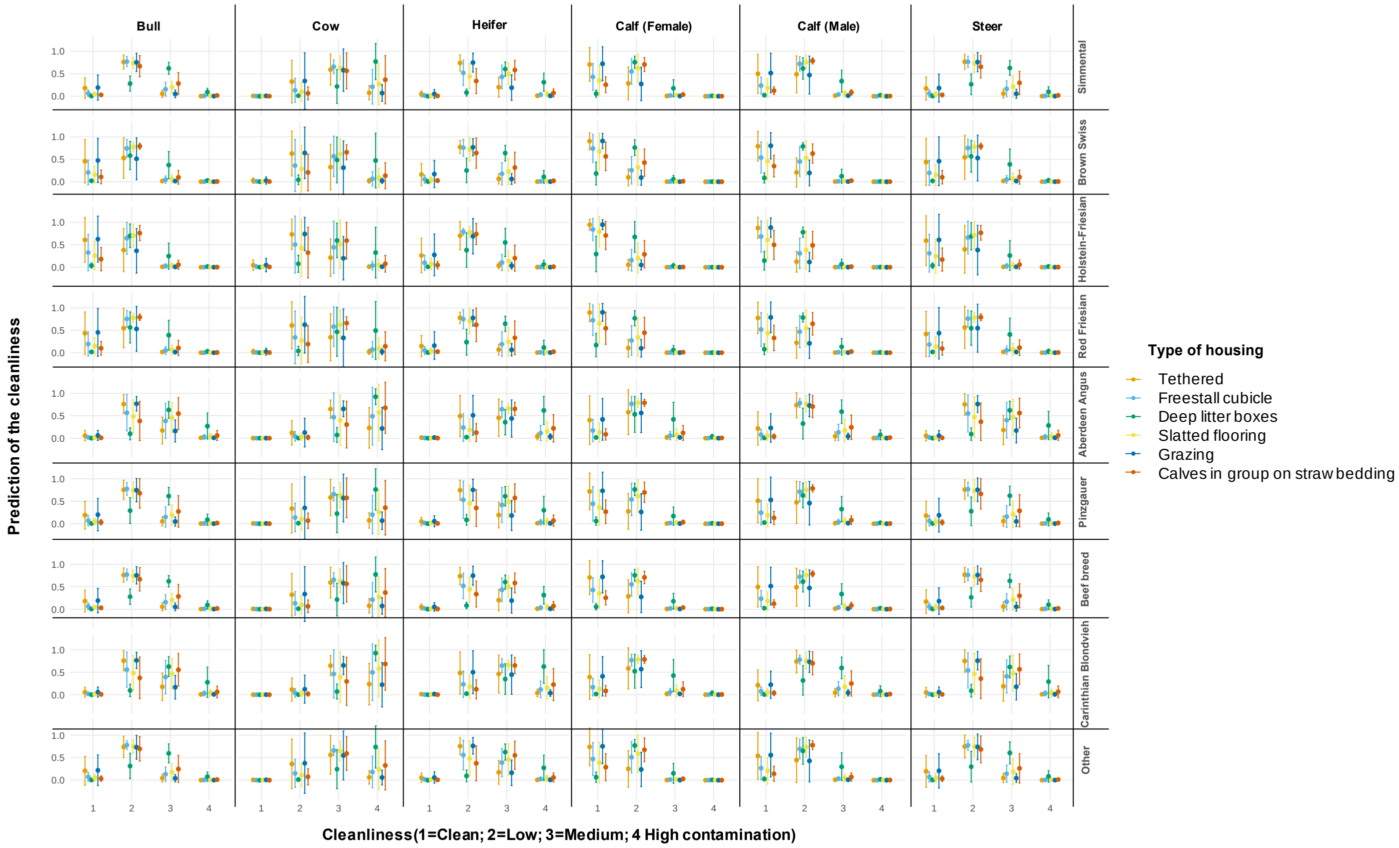

Figure S3. The expected probability (range: 0-1) for a cleanliness level depending on type of housing stratified by sex and breed of the slaughtered cattle.
